# MOF-Derived Ni_1−*x*_Co_*x*_@Carbon with Tunable Nano–Microstructure as Lightweight and Highly Efficient Electromagnetic Wave Absorber

**DOI:** 10.1007/s40820-020-00488-0

**Published:** 2020-07-15

**Authors:** Lei Wang, Mengqiu Huang, Xuefeng Yu, Wenbin You, Jie Zhang, Xianhu Liu, Min Wang, Renchao Che

**Affiliations:** 1 Laboratory of Advanced Materials, Department of Materials Science and Collaborative Innovation Center of Chemistry for Energy Materials (iChem), Fudan University, Shanghai, 200438 People’s Republic of China; 2 Key Laboratory of Materials Processing and Mold (Zhengzhou University), Ministry of Education, Zhengzhou, 450002 People’s Republic of China

**Keywords:** Metal–organic frameworks, Polarization, Magnetic coupling, Microwave absorption, Electromagnetic parameters

## Abstract

Intrinsic electric-magnetic property and special nano-micro architecture of functional materials have a significant effect on its electromagnetic wave energy conversion, especially in the microwave absorption (MA) field. Herein, porous Ni_1−*x*_Co_*x*_@Carbon composites derived from metal-organic framework (MOF) were successfully synthesized via solvothermal reaction and subsequent annealing treatments. Benefiting from the coordination, carbonized bimetallic Ni-Co-MOF maintained its initial skeleton and transformed into magnetic-carbon composites with tunable nano-micro structure. During the thermal decomposition, generated magnetic particles/clusters acted as a catalyst to promote the carbon *sp*^2^ arrangement, forming special core-shell architecture. Therefore, pure Ni@C microspheres displayed strong MA behaviors than other Ni_1−*x*_Co_*x*_@Carbon composites. Surprisingly, magnetic-dielectric Ni@C composites possessed the strongest reflection loss value − 59.5 dB and the effective absorption frequency covered as wide as 4.7 GHz. Meanwhile, the MA capacity also can be boosted by adjusting the absorber content from 25% to 40%. Magnetic–dielectric synergy effect of MOF-derived Ni_1−*x*_Co_*x*_@Carbon microspheres was confirmed by the off-axis electron holography technology making a thorough inquiry in the MA mechanism.

**Electronic supplementary material:**

The online version of this article (10.1007/s40820-020-00488-0) contains supplementary material, which is available to authorized users.

## Highlights

- MOF-derived porous Ni_1−*x*_Co_*x*_@Carbon composites with tuning nano-micro structure were successfully synthesized.
- Magnetic-dielectric synergy effect among the Ni_1−*x*_Co_*x*_@Carbon microspheres was confirmed by the off-axis electron holography technology.
- MOF-derived Ni@C microspheres displayed strong microwave absorption value of − 59.5 dB..

**Electronic supplementary material:**

The online version of this article (10.1007/s40820-020-00488-0) contains supplementary material, which is available to authorized users.

## Graphical abstract

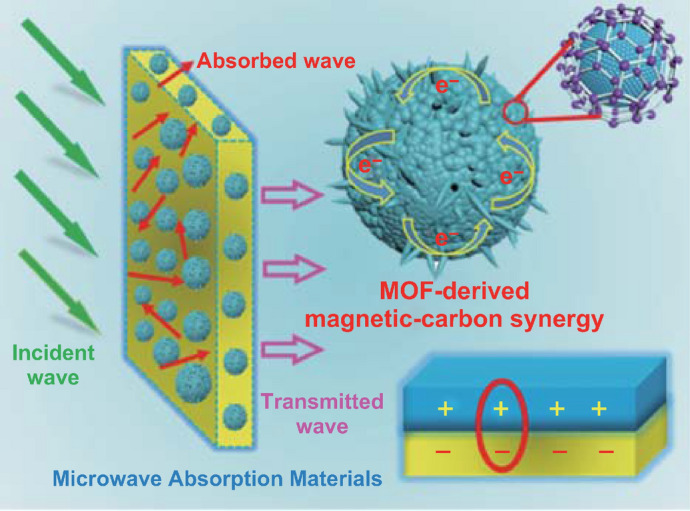

## Introduction

Metal–organic frameworks (MOF), constructing by inorganic metal ion and organic linkers via strong chemical bonds, have gained rapid development and huge influence in the past 2 decades. As functional materials, MOF possess versatile advantages including multi-dimension morphology, specific structure, high surface area, controllable pore size, and so on [1–4]. Based on these fascinating properties, MOF have displayed its broad application prospects in various fields, such as energy catalysis, gas storage/separate, solar battery, and biomedicine [5–7]. More interestingly, to obtain the desired chemical/physical characteristic, MOFs can also be used as the precursor or template to fabricate target materials. By applying different treatment methods and modification, plenty of MOF derivatives with unique electromagnetic behaviors were smartly designed [8]. For instance, Yamauchi et al. [9] reported a selectively nanoporous hybrid carbon from core–shell structured ZIF-8@ZIF-67 crystals, which exhibits a distinguished specific capacitance. Hu et al. [10] utilized a general MOF-derived selenidation strategy to synthesize in situ carbon-encapsulated selenides, these selenides with particular nano–micro-structured features and ultrastable cycling performance as Na-ion batteries. Rosei et al. [11] designed a MOF-derived TiO_2_ photoanodes sensitized with quantum dots creating a favorable band energy alignment for the separation of the photogenerated charges. As a result, MOF-derived materials have drawn wide research as functional materials because of fine structure regulation and selective preparation. At the same time, building a composite structure with other active materials and constructing a special morphology can further strengthen its role in practical applications.

For microwave absorption (MA) materials, the complex permittivity and permeability occupy a vital position, which determines material capacity to store and loss electromagnetic waves energy. Generally, the MA performance is mightily governed by the overall effect of the intrinsic electrical and magnetic characteristics modulating by component, dimensional, electron conductivity, and electromagnetic balance [12–15]. Due to facile synthesis process, adjustable components, controllable shape, and suitable electromagnetic properties, MOF-derived MA materials have become research hotspots. In recent years, plenty of MOF derivatives materials firstly focus on the magnetic–dielectric composites with broadband absorption and strong reflection loss (RL). Ji et al. [16] fabricated a MOF-derived Co/C composite through interface design with enhanced low-frequency electromagnetic properties. Xu [17] group reported MOF-derived hollow Co/C microspheres with enhanced microwave absorption performance. Che et al. [18] designed similar “Schottky contact” in the MOF-derived yolk–shell Ni@C@ZnO absorber, which exhibits excellent MA performance. Yu et al. [19] synthesized MOF-derived porous Fe/C composite as a lightweight and highly efficient electromagnetic wave absorber.

Secondly, to prove the dielectric loss ability and impedance matching, hybridizing the MOF derivatives and high conductive substrate proved an effective solution. Yu et al. [20] used multi-walled carbon nanotubes as templates for growth of Co-based zeolitic imidazolate frameworks and obtained a Co–C/MWCNTs composite. Lv et al. [21] integrated ZnO/NPC/RGO samples derived from the MOF/RGO hybrid with tunable dielectric performance. He et al. [22] rationally constructed Co/TiO_2_–C composites for enhanced polarization behaviors and boosted conductivity loss starting from MXene/MOF hybrids. In adding, benefiting from the diversity of coordination between the organic and inorganic units, MOFs can be fabricated into changeable dimensional and sculptured into specific structure. Zhang et al. [23] achieved MOF-derived one-dimensional (1D) porous ZnO/C nanofiber with lightweight and enhanced microwave response by an electrospinning method. Hou et al. [24] obtained a MOF-derived three-dimensional (3D) nanoporous carbon composite toward the electromagnetic functionalization. Meanwhile, MOF-derived MA composites have extremely changeable morphology, such as cube-like Fe/C, rambutan-like C/nanotubes/Co composites, polyhedral-like CuO/C composites, honeycomb-like Co/C composites, and flower-like Ni/C composites [25–29]. The above-mentioned factors all will influence the electron transformation and magnetic responding, and its final MA performance may be changed because of those structure regulation. Obviously, electromagnetic parameters have always been the focus of research to obtain excellent microwave absorbing materials, which faces huge challenges. In addition, the inherent magnetic loss mechanism of MA materials is still unclear and needs to be further explored.

Herein, using the Ni–Co–MOF as the template, large-sized MOF-derived Ni_1−*x*_Co_*x*_@Carbon (Ni@C) composites with tuning nano–microstructure are successfully synthesized via combining the solvothermal reaction and carbonization treatment under Ar atmosphere. Considering the coordination effect between metal elements and inorganic linkers, we project an investigation on the MA ability of a series of Ni@C composites, which construct with different Ni/Co adding ratios. Reduced Ni/Co particles/clusters acted as a catalyst further to facilitate the formation of graphitized carbon layers, which wrap the inner magnetic and build a special core–shell unit. The related results demonstrated that Ni@C composites exhibit increased saturation magnetization (*M*s) value, higher conductivity, and enhanced RL ability with the nickel increased nickel content. Therefore, strong magnetic Ni@C microspheres show strongest MA capacity than other Ni@C absorbers. Surprisingly, special Ni@C microspheres have optimized RL value up to − 59.6 dB at only 25% adding. Meanwhile, the effective absorption frequency of magnetic–dielectric Ni@C covers as wide as 4.7 GHz ranging from 9.9 to 14.6 GHz. To probe the intrinsic electromagnetic property and magnetic loss mechanism, off-axis electron holography technology was applied to prove the polarization behavior at the interfaces and magnetic coupling between particles.

## Experimental Section

### Synthesis of Magnetic Ni@C Microspheres

All of the chemicals used were of analytical grade without further purification and were purchased from Sinopharm Chemical Reagent Co., Ltd. In a typical synthesis, Co(NO_3_)_2_·6H_2_O, Ni (NO_3_)_2_·6H_2_O, 0.15 g *p*-benzenedicarboxylic acid, and 1.0 g PVP K-30 were dissolved in the mixture solution, which contains deionized water (10 mL), ethanol (10 mL), and N, *N*-dimethylformamide (10 mL). The obtained solution was intensely magnetic stirred for 30 min and transferred to a 50-mL autoclave and keep at 150 °C for 12 h. The gained Co–Ni–MOF products are collected via centrifugation and washing. As-prepared dried products are heated at 600 °C under Ar flow for 5 h with a heating rate of 2 °C min^−1^. To investigate the effect of different Co^2+^/Ni^2+^ addition ratios on the nano–microstructure and electromagnetic properties, Co(NO_3_)_2_·6H_2_O and Ni (NO_3_)_2_·6H_2_O were added into the solution following millimole content: M_Ni_/M_Co_ = 1.5/0, 1.2/0.3, 0.75/0.75, 0.3/1.2, and 0/1.5, respectively. The final magnetic Ni_1−*x*_Co_*x*_@Carbon powders were marked as Ni@C, Ni_0.8_Co_0.2_@C, Ni_0.5_Co_0.5_@C, Ni_0.2_Co_0.8_@C, and CoO@C, respectively.

### Characterizations

The chemical composition of the final products is characterized via powder X-ray diffractometer (XRD, Bruker, D8-Advance, Germany). Raman data are obtained on a Ramoscope with a He–Ne laser (inVia, Renishaw, UK). The magnetic prosperities of Ni_1−*x*_Co_*x*_@Carbon microspheres are measured by vibrating sample magnetometer (MPMS (SQUID) VSM, Quantum Design, USA). The morphology and microstructure of the magnetic powders are examined by a field-emission scanning electron microscope (FESEM, S-4800, Japan) and a field-emission transmission electron microscope (TEM, JEM-2100F, Japan). The absorber samples are prepared by uniformly mixing the magnetic powders with paraffin matrix in different mass ratios 25% and 40%. The sample is compacted into a coaxial ring of 7.00 mm outer diameter and 3.04 mm inner diameter. Related electromagnetic parameters were measured via a vector network analyzer (VNA, HP8510C, Agilent, USA) over the 2–18 GHz range. Related reflection loss curves are calculated by the following equations [30, 31]:

1 $$Z_{\text{in}} = Z_{0} \sqrt {\left( {\mu_{r} /\varepsilon_{r} } \right)} \tanh \left[ {j\left( {\frac{2\pi fd}{c}} \right)\sqrt {\mu_{r} \varepsilon_{r} } } \right]$$

2 $${\text{RL}} = 20\log \left| {\left( {Z_{\text{in}} - Z_{0} } \right)/\left( {Z_{\text{in}} + Z_{0} } \right)} \right|$$

where *Z*_in_ and *Z*_0_ are the input characteristic impedance and the free space impedance, *ε*_*r*_ is the complex permittivity and *μ*_*r*_ is the complex permeability, ƒ is the tested frequency at 2–18 GHz, d is the simulation thickness, c is the velocity of microwave in free space, respectively.

## Results and Discussion

### Fabrication and Characterization of Ni@C Composites

**Fig. 1:**
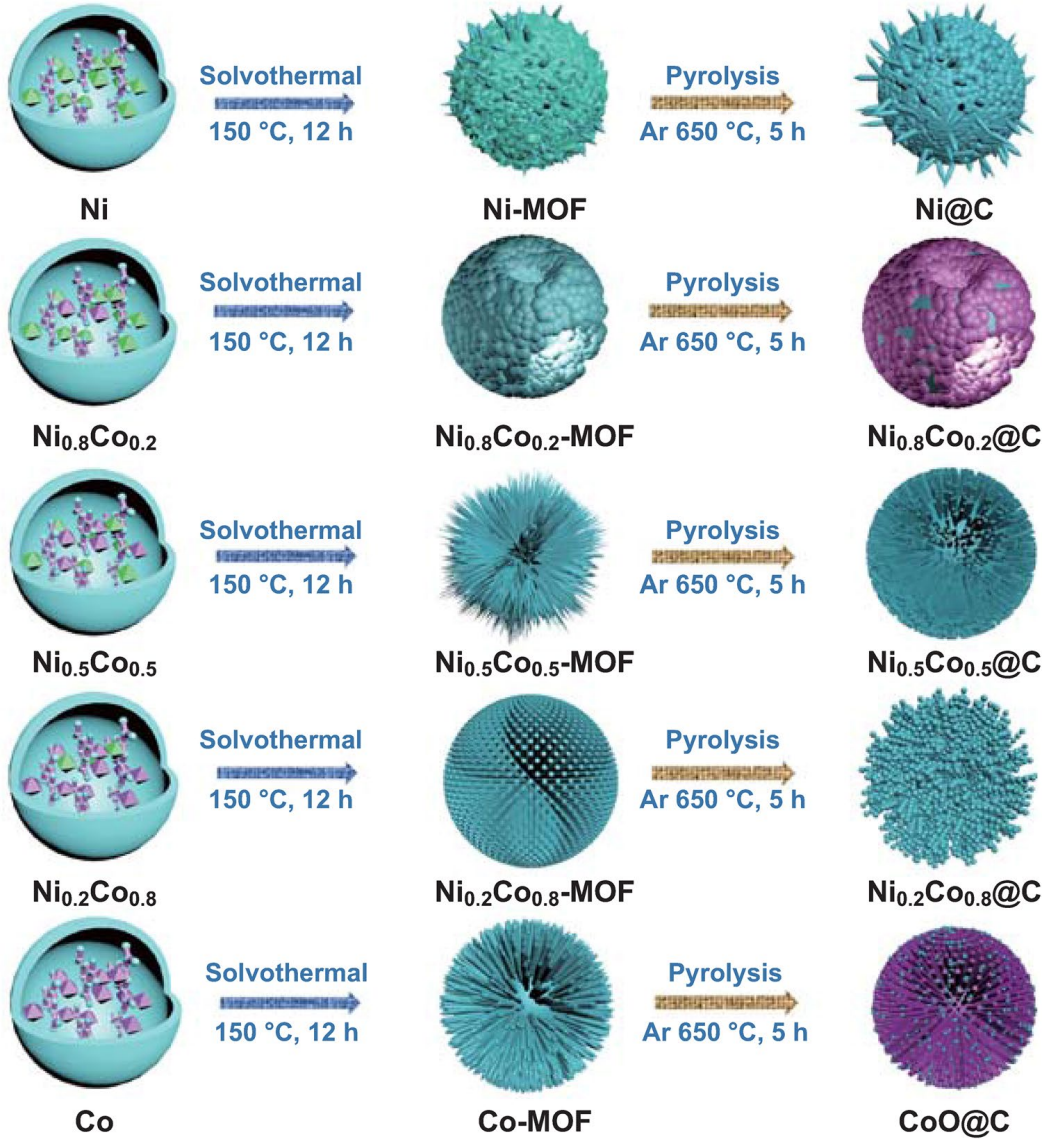
Schematic illustration for the synthetic process of Ni_1−*x*_Co_*x*_@Carbon microspheres

**Fig. 2:**
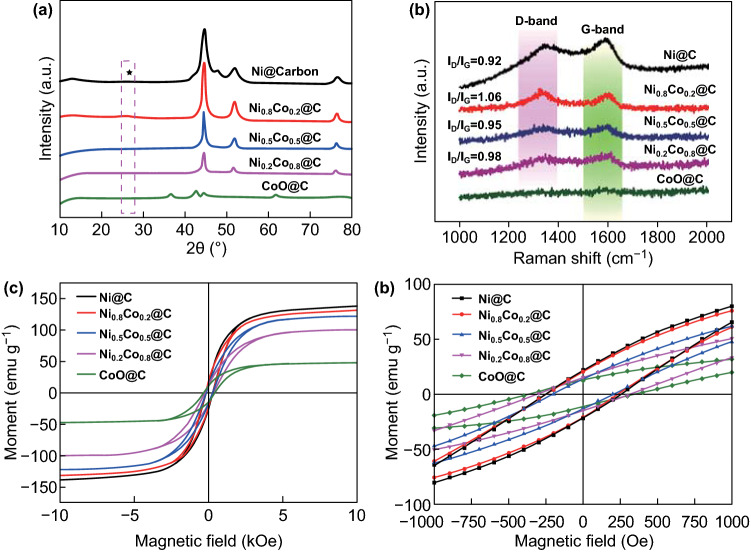
**a** XRD patterns, **b** Raman spectrum, **c**, **d** magnetic hysteresis loops of the Ni@C composites

Porous Ni@C microspheres are fabricated via a facile two-step process as displayed in Fig. 1. A series of Ni–Co–MOF precursors are firstly obtained after a solvothermal reaction in the mixed H_2_O/DMF/EtOH solution. Due to the space coordination effect between metal ions and linkers, Ni–Co–MOF precursors show changeable nano–microstructure and increased particle size. MOF-derived Ni@C exhibited a similar increase size distribution (Table S1). Before obtaining the Ni@C microspheres, the Ni–Co–MOF templates were carbonized in the given Ar atmosphere. Reduced metal/alloy particles can be work as the catalysis to encourage the rearrangement of carbon element in the bridged organic ligands (Fig. 1). Limited by the original periodic network structure, the MOF-derived Ni@C composites kept similar MOF framework exhibiting different components and materials distribution. As shown in Fig. 2a, the XRD patterns clearly demonstrated that the magnetic powders are Ni@C, Ni_0.8_Co_0.2_@C, Ni_0.5_Co_0.5_@C, Ni_0.2_Co_0.8_@C, and CoO@C, respectively. Even undergoing equal annealing environment, adjustable catalytic ability of transformed magnetic Ni or NiCo alloy can be efficiently regulated by increasing Ni/Co content ratio. It is worth noting that the carbonized Co–MOF is consisted of CoO particles and carbon matrix without the cobalt metal (Fig. S1).

Then, the Ni@C composites exhibited controllable electromagnetic properties accompanied by the enhanced saturation magnetization and increased graphitized carbon content. In Fig. 2b, there are two characteristic Raman peaks of the carbon materials in the obtained Ni@C composites, which locates at 1339.4 cm^−1^ (D-band) and 1592.1 cm^−1^ (G-band), respectively. Generally, the D-band represents the disorder carbon structure and the atomic crystal defects, and the other G-band means the in-plane stretching vibration stemming from the *sp*^2^ hybrid carbon atom. Therefore, the intensity ratio of *I*_D_/*I*_G_ is used as a referenced standard to evaluate the graphitized degree of carbon component. As Cobalt adding content increases, the values of *I*_D_/*I*_G_ of obtained magnetic powders reflect a downward trend from the Ni@C microspheres to the CoO@C composites. The intensity data of Ni@C, Ni_0.8_Co_0.2_@C, Ni_0.5_Co_0.5_@C, and Ni_0.2_Co_0.8_@C composites are 0.92, 1.06, 0.95, and 0.98, respectively. Due to the poor catalytic ability in the thermal treatment process, as-synthesized CoO@C material exhibits weak D-band and G-band signal.

Simultaneously, magnetic properties of micro-size Ni@C composites are tested at room temperature and related results are pictured in Fig. 2c. From the magnetic hysteresis (*M* vs *H*) loop, the saturation magnetization (*M*s) value of Ni@C, Ni_0.8_Co_0.2_@C, Ni_0.5_Co_0.5_@C, Ni_0.2_Co_0.8_@C, and CoO@C is 138.5, 131.4, 121.1, 100.7, and 47.2 emu g^-1^, respectively. Magnetic Ni@C composite has the highest *M*s value, which indicates strongest storage for the microwave energy in terms of magnetic component. Compared with the CoO@C microspheres, the other three samples still exhibit better magnetic response capacity, which is necessary to the enhanced magnetic loss ability. Among the magnetic features, the coercivity force (*H*c) plays a significant factor to determine the hysteresis and magnetic permeability. The Hc values of five composites are 195, 248, 250, 305, and 356 Oe, respectively. Increased *H*c values from Ni@C to CoO@C microsphere are highly associated with the magnetic particles size, space distribution, and integrated responding behavior. According to the latest published literatures, promoted magnetic permeability is benefit to the electromagnetic wave energy conversion because of the higher *M*s and suitable *H*c values [32–34].

**Fig. 3:**
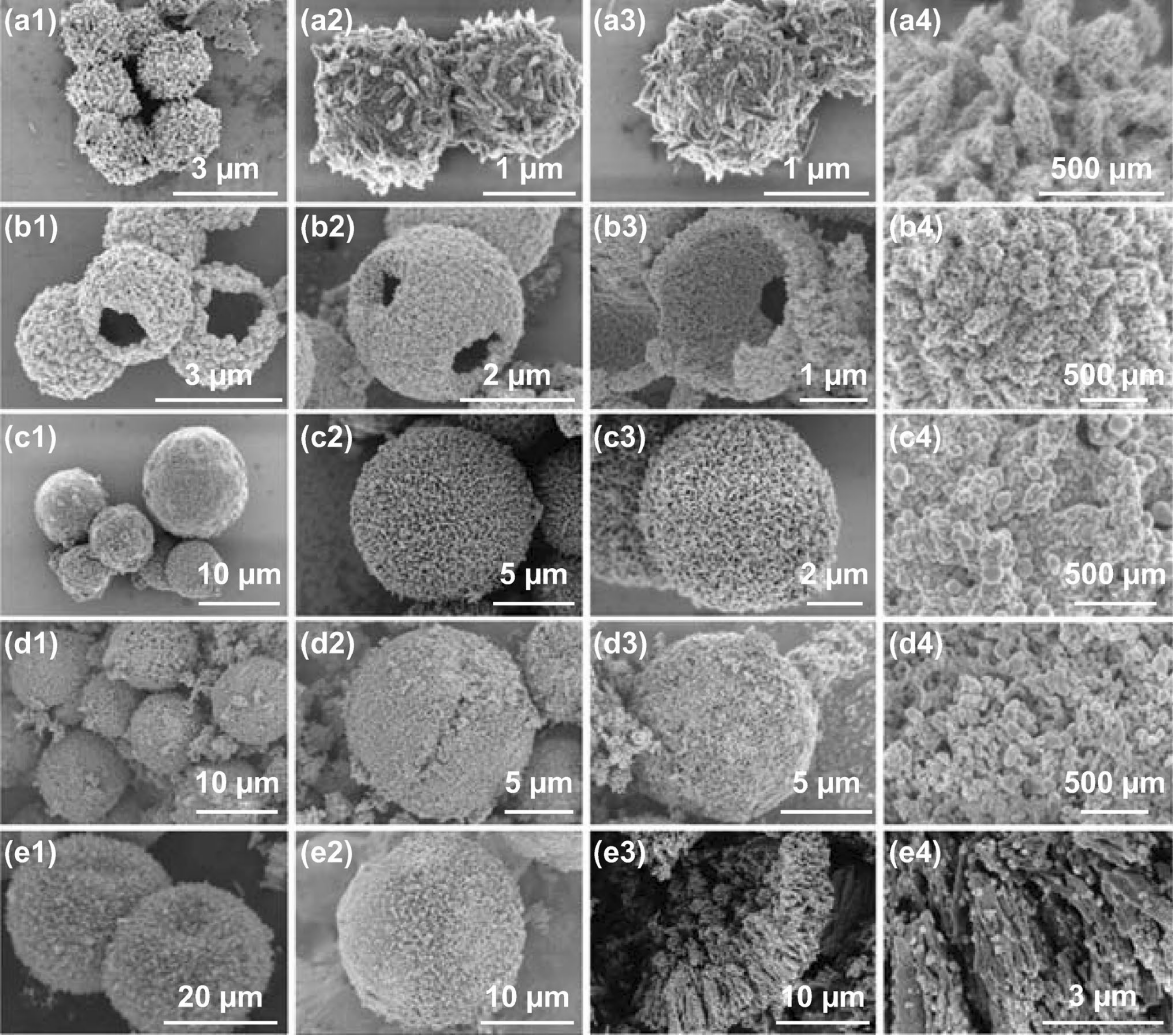
SEM images of **a1–a4** Ni@C, **b1–b4** Ni_0.8_Co_0.2_@C, **c1–c4** Ni_0.5_Co_0.5_@C, **d1–d4** Ni_0.2_Co_0.8_@C, and **e1–e4** CoO@C composites

The morphology and microstructure of MOF-derived porous Ni@C composites are investigated and displayed in Fig. 3. The obtained Ni@C microspheres have a particles size of 1.5–2.0 μm, which is assembled by the micro-size spheres and attached spindle-like particles (Fig. 3a1–a4). Different with the Ni@C materials, other four samples showed uniform sphere structure and increased particles size. In Fig. 3b, monodisperse Ni_0.8_Co_0.2_@C microspheres have a special hollow structure and uniform size distribution about ~ 3 μm (Fig. 3b1–b4). For the Ni_0.5_Co_0.5_@C composites, carbonized particles maintain similar micro-scale framework compared with original MOF precursor. MOF-derived Ni_0.5_Co_0.5_@C material possesses larger nanoscale unit and sphere size ~ 8 μm (Fig. 3c1–c4). When the Ni_0.2_Co_0.8_–MOF is converted into the Ni_0.2_Co_0.8_@C composites, both the size and architecture of obtained microspheres reveal slight variation (Fig. 3d1–d4). There are many tiny particles anchored on surface of the microspheres, which has an increasing size about ~ 10 μm. Without the existence of Ni metal, pure Co-MOF powder displays a unique growth orientation and determines the final shape of MOF derivatives. As shown in Fig. 3e, obtained CoO@C composites reflect the largest size distribution ~ 30 μm and consist of parallel and radial columnar carbon framework (Fig. 3e1–e3). Generated CoO nanoparticles randomly decorate on the carbon matrix without agglomeration (Fig. 3e4). Related elements mapping distribution of as-synthesized Ni@C is depicted in Fig. S2.

**Fig. 4:**
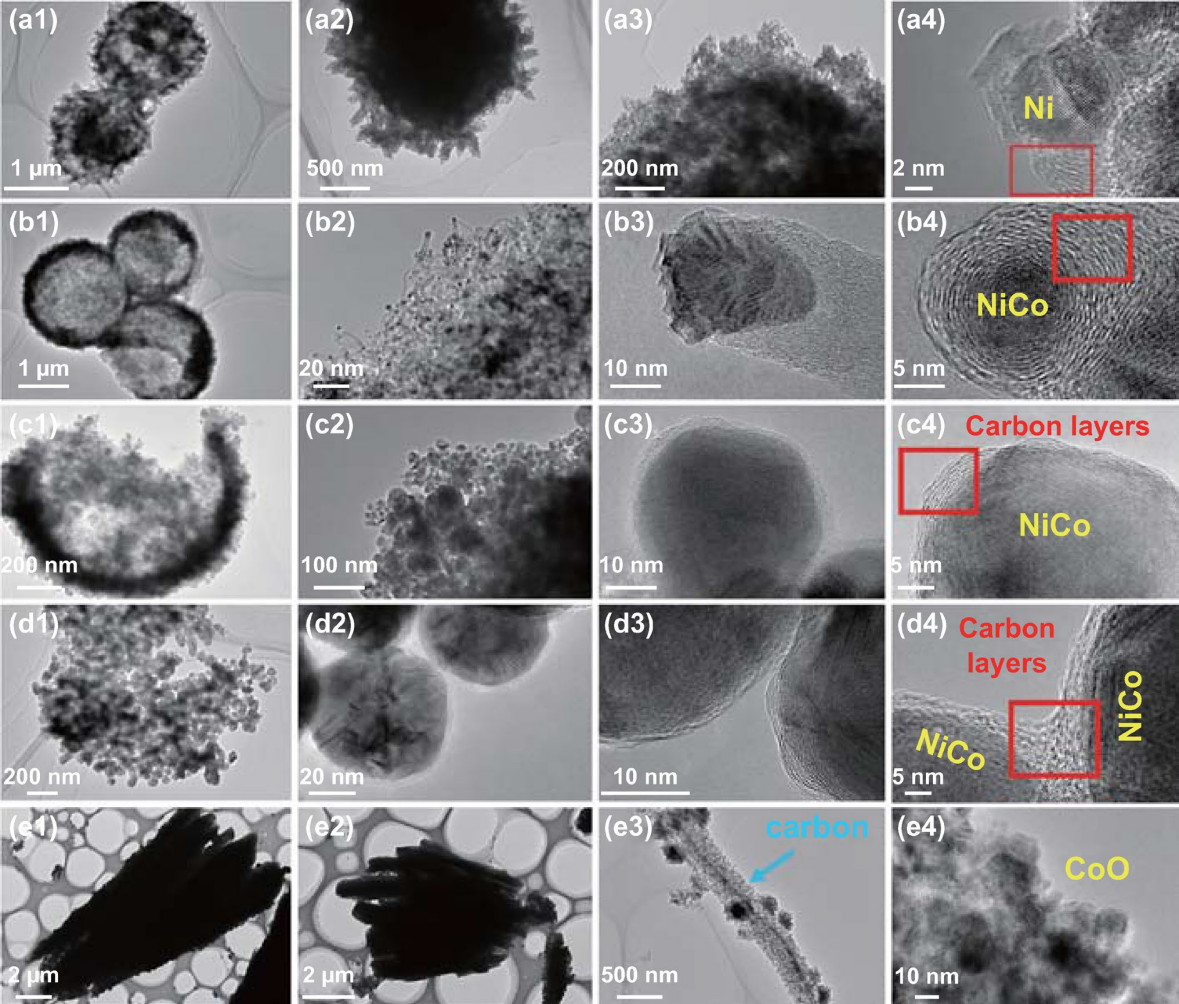
TEM images of **a1–a4** Ni@C, **b1–b4** Ni_0.8_Co_0.2_@C, **c1–c4** Ni_0.5_Co_0.5_@C, **d1–d4** Ni_0.2_Co_0.8_@C, and **e1–e4** CoO@C composites

To confirm the distribution and microstructure of the magnetic carbon composites, all the as-synthesized MOF-derived composites at micrometer scale are characterized by the TEM and related results are pictured in Fig. 4. Combined with the SEM images, the TEM images can give a direct and clear observation to the component distribution, especially the location of the magnetic substance. Signal Ni@C microsphere has a hierarchical structure and many nanoscale magnetic carbon basic units (Fig. 4a1–a3). Clearly, the graphited carbon layers tightly wrap the metal particles, meaning that the Ni substance has high catalytic activity during carbon *sp*^2^ arrangement process (Fig. 4a4). After carbonized the Ni_0.8_Co_0.2_–MOF precursor, porous Ni_0.8_Co_0.2_@C composites show a unique hollow structure and those derivatives accumulate in the shell (Fig. 4b1). Further magnifying the surfaces of the Ni_0.8_Co_0.2_@C microspheres, plenty of short carbon nanotubes grow from the inside of the ball and entangle each other (Fig. 4b2). Part of the NiCo alloy particles are encapsulated in the top of the carbon tube, and the rest magnetic cores are enclosed by the transformed high conductivity carbon layers (Fig. 4b3, b4). For the Ni_0.5_Co_0.5_@C composites, they display an increased size property both the microspheres itself and the magnetic NiCo alloy (Fig. 4c1–c2). As the shown in the pictures, the size of the NiCo alloy is larger about diameters of ~ 50 nm (Fig. 4c3, c4). Meanwhile, the carbon layers’ number exhibits a slight downtrend. Similarly, MOF-derived Ni_0.2_Co_0.8_@C composite is assembled by lots of connecting magnetic carbon unit, constructing a porous three-dimensional microsphere and keeping original MOF skeleton (Fig. 4d1–d4). As mentioned before, obtained CoO@C possesses a remarkable parallel and radial columnar carbon framework with spaced channels (Fig. 4e1–e3). From the single carbon substrate, randomly distributed CoO particles anchored into the carbon matrix and the CoO fully exposed to space, which demonstrate a huge distinguish with others carbonized MOF derivative (Fig. 4e4). As we know, the components, morphology, magnetic properties, and conductivity all have a significant impact on the final electromagnetic parameters and microwave absorption performance [35–37].

### Electromagnetic Parameters Analysis and Microwave Absorption Ability

**Fig. 5:**
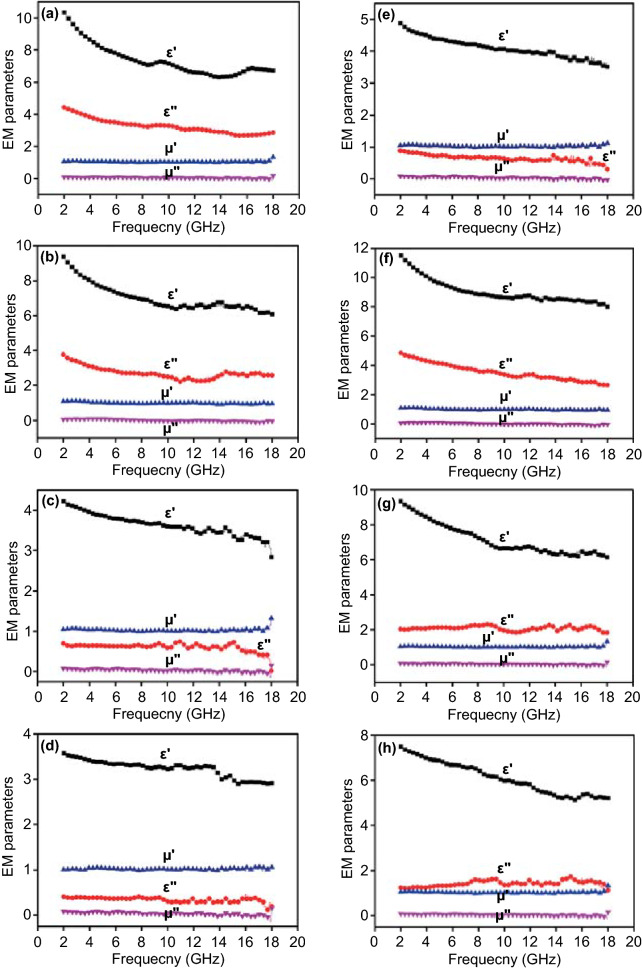
Electromagnetic parameters of **a**, **e** Ni@C, **b**, **f** Ni_0.8_Co_0.2_@C, **c**, **g** Ni_0.5_Co_0.5_@C, and **d**, **h** Ni_0.2_Co_0.8_@C composites at 25%, and 40% mass adding, respectively

In order to investigate the intrinsic electromagnetic characteristics of obtained MOF-derived black magnetic powders, the electromagnetic parameters are measured by the vector network analyzer (VNA) according the transmitted line theory [38–40]. As shown in Fig. 5, the relationship between tested data and the frequency is plotted to demonstrate the complex permittivity (*ε*_r_ = *ε*′ − *jε*″) and the relative complex permeability (*μ*_r_ = *μ*′ − *jμ*″) from 2 to 18 GHz. As the MA theory said, the real parts (*ε*′, *μ*′) imply the storage electrical and magnetic capacity toward the incident microwave energy, while the imaginary parts (*ε*″) represent the matched dissipation ability to the electrical and magnetic components [41, 42]. When the mass adding is 25%, the real parts of complex permeability reflect a typical frequency dependence property, which depicts a decreasing tendency from the low frequency to the high frequency region. The *ε*′ values range from 10.4 to 6.8 for Ni@C, from 9.4 to 6.1 for Ni_0.8_Co_0.2_@C, from the 4.2 to 3.0 for Ni_0.5_Co_0.5_@C, and from 3.6 to 2.8 for Ni_0.2_Co_0.8_@C, respectively. Among those MA composites, pure Ni@C composite has the highest *ε*′ value meaning the strongest ability for storing the electromagnetic wave energy. Similarly, the imaginary part (*ε*″) values exhibit a decreasing curve, falling from 4.4 to 2.8 for Ni@C, from 3.8 to 2.6 for Ni_0.8_Co_0.2_@C, from 0.7 to 0.3 for Ni_0.5_Co_0.5_@C, and from 0.4 to 0.2 for Ni_0.2_Co_0.8_@C, respectively. The real part (*μ*′) and imaginary part (*μ*″) of complex permeability of obtained Ni@C composites display a similar value, which keeps at ~ 1.1 of *μ*′ values and at ~ 0.08 of *μ*″, respectively (Fig. 5). By comparing these MA samples, it can be found that the increased cobalt content introduces weakened electromagnetic parameters at 25% mass adding.

To adjust the MA performance, the mass adding content increases from 25 to 40% for the better understanding mass factor. Obviously, the electromagnetic parameters make a significant difference, especially in the complex permittivity. As displayed in Fig. 5a, e, both the storage and dissipation ability of Ni@C composites exhibit a sharp decline. The *ε*′ values drop by half and the *ε*″ data reach very low level, which is not benefit to the energy conversion [43]. Inspiringly, other MOF-derived Ni@C composites show a rising tendency after adding more mass components, which indicates the boosted loss behaviors (Fig. 5b, f). It is worth noting that the *ε*′ values start from 11.5 to 8.0 for Ni_0.8_Co_0.2_@C, from 9.4 to 6.2 for Ni_0.5_Co_0.5_@C, and from 7.5 to 5.2 for Ni_0.2_Co_0.8_@C (Fig. 5b–d, f–h). For the Ni@C composites with strong magnetic properties and high electronic conductivity, the 25% adding mass is the suitable condition for the absorber/paraffin system. After adding more Ni@C powders into mixed absorber, it will break the continuous dielectric system and weaken the requirement of impedance matching, leading more incident microwave reflecting black to the space rather than penetrating into the absorber. As expected, hollow Ni_0.8_Co_0.2_@C composites bring a remarkable promotion both in the real part (*ε*′) and imaginary part (*ε*″), encouraging the amelioration of electromagnetic energy conversion. At the same time, the same upward trend of electromagnetic parameters is also seen in porous Ni_0.5_Co_0.5_@C and Ni_0.2_Co_0.8_@C composites. In adding, related electromagnetic properties of MOF-derived CoO@C composites are studied and discussed at different adding mass conditions. Unfortunately, no matter how the content of CoO@C powders is adjusted, there is no change in its fundamental electromagnetic properties. Large-sized CoO@C microspheres assembled by CoO particles and amorphous carbon cannot absorb the incident microwave energy reflected by the responding parameters (Fig. S3). As a result, the derivative CoO@C from carbonized original Co-MOF precursor is unsuitable for applications in the MA field. As above discussed, adjusted electromagnetic behavior of MOF-derived magnetic carbon absorbers is contributed to the changeable magnetic components, spatial distribution, and induced dielectric properties.

**Fig. 6:**
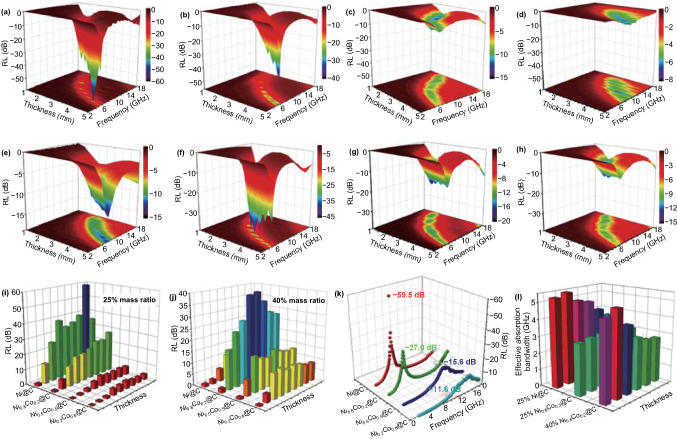
3D RL map of **a**, **e** Ni@C, **b**, **f** Ni_0.8_Co_0.2_@C, **c**, **g** Ni_0.5_Co_0.5_@C, and **d**, **h** Ni_0.2_Co_0.8_@C composites at 25%, and 40% mass adding, respectively. **i**, **j** 3D histogram of RL values, **k** RL curves, and **l** 3D histogram of EAB of Ni@C composites

Based on the obtained electromagnetic parameters and transmission line theory, related reflection loss (*RL*) curves of as-synthesized MOF-derived Ni_1−*x*_Co_*x*_@Carbon composites are calculated and displayed in Fig. 6. Those magnetic–dielectric composites clearly embody the adjustment and optimization of MA performance reflected by the three-dimensional (3D) RL maps. When the adding mass is 25%, the porous Ni@C absorber describes the excellent MA behavior including the RL values and effective absorption region. As displayed in Fig. 6a, as the frequency increases, the RL values of as-synthesized magnetic–dielectric system show a visible variation at different thicknesses, which is consistent with the traditional and classic MA theory. Altering the simulated absorbers’ thickness, a series of peak values can be received and the maximum RL values of Ni@C composites are − 13.1, − 25.8, − 39.1, − 33.8, − 35.6, − 39.4, − 59.5, and − 36.4 dB from the 1.5 to 5.0 mm, respectively. The maximum *RL* value of Ni@C can reach up to − 59.5 dB at the C-band, implying the strongest loss ability (Fig. 6a). Simultaneously, those absorption peaks values shift from the Ku-band to the S-band with increasing thickness. For the hollow Ni_0.8_Co_0.2_@C composites, the maximum *RL* value reaches to − 27.0 dB at 5.7 GHz at 5.0 mm (Fig. 6b). Compared with the above-mentioned two samples, Ni_0.5_Co_0.5_@C and Ni_0.2_Co_0.8_@C composites possess lower value of − 6.8 dB at 10.9 GHz and − 6.4 dB at 13.9 GHz, revealing the lower MA intensity at same quality ratio (Fig. 6c, d, k). By increasing the adding mass to 40%, the MA capacity of MOF-derived Ni@C composites has been greatly improved except for pure Ni@C composites. The maximum RL values promote from − 27.0 to − 39.3 dB at 3.5 mm for Ni_0.8_Co_0.2_@C, from − 6.8 to − 15.6 dB at 2.0 mm for Ni_0.5_Co_0.5_@C, and from − 6.4 to − 11.6 dB at 2.5 mm for Ni_0.2_Co_0.8_@C, respectively, manifesting the thin applied thickness and boosted microwave loss ability (Fig. 6f–h). Due to the poor impedance matching from excessive addition, Ni@C absorber does not show enhanced MA behavior as expected (Fig. 6e).

To evaluate the MA capabilities of obtained Ni@C composites, the effective absorption frequency is another important indicator [44, 45]. Generally, it means that 90% electromagnetic wave energy can be dissipated when the *RL* value up to − 10 dB. At the same time, the corresponding frequency scale, RL ≤ − 10 dB, is defined as effective absorption bandwidth (EAB). As the adding mass is 25%, the EAB of porous Ni@C composite covers as wide as 4.7 GHz ranging at 2.5 mm and 4.9 GHz ranging at 2.2 mm (Fig. 6l). Hollow Ni_0.8_Co_0.2_@C absorber exhibits a narrow EAB because of the inferior electromagnetic energy conversion compared with the Ni@C composites at same adding quality. Enhanced the adding mass to the 40%, the EAB and the *RL* intensity of Ni_0.8_Co_0.2_@C composite gets a huge improvement (Fig. 6i–l). It can be seen that EAB expands from initial 3.3 to 4.8 GHz, stemming from the strengthened microwave responding ability and dissipation intensity (Fig. 6f). Meanwhile, the efficient absorption areas of obtained Ni@C composites are greatly improved (Fig. S4). After comprehensive comparison, MOF-derived Ni@C absorber possesses the stronger RL value (− 59.5 dB), wider EAB (4.7 GHz), and lower adding mass (25%), which is favor to be a lightweight and an excellent MA material. To prove the MA performance of other MOF derivatives, increasing the absorber content is a valuable strategy to solute the limited RL and microwave loss ability. As mentioned above, MOF-derived kinds of Ni@C composites exhibit excellent electromagnetic storages and microwave absorption capacities, resulting in the enhanced reflection loss values and expanded effective absorption frequency. Among those absorbers, strong magnetic Ni@C composites have the powerful MA ability with the maximum *RL* value of − 59.5 dB and 4.7 GHz of EAB at 25% adding mass. By increasing the absorber content, the MA performance of other Ni@C composites gets a substantial amelioration. Hollow Ni_0.8_Co_0.2_@C possesses a *RL* value of − 39.3 dB and an increased EAB of 4.8 GHz at 40% adding mass. Compared with original MA characteristics, the *RL* values of high content Ni_0.5_Co_0.5_@C and Ni_0.2_Co_0.8_@C composite have nearly doubled meaning the promoted MA performance.

### Analysis of Microwave Absorption Mechanism

**Fig. 7:**
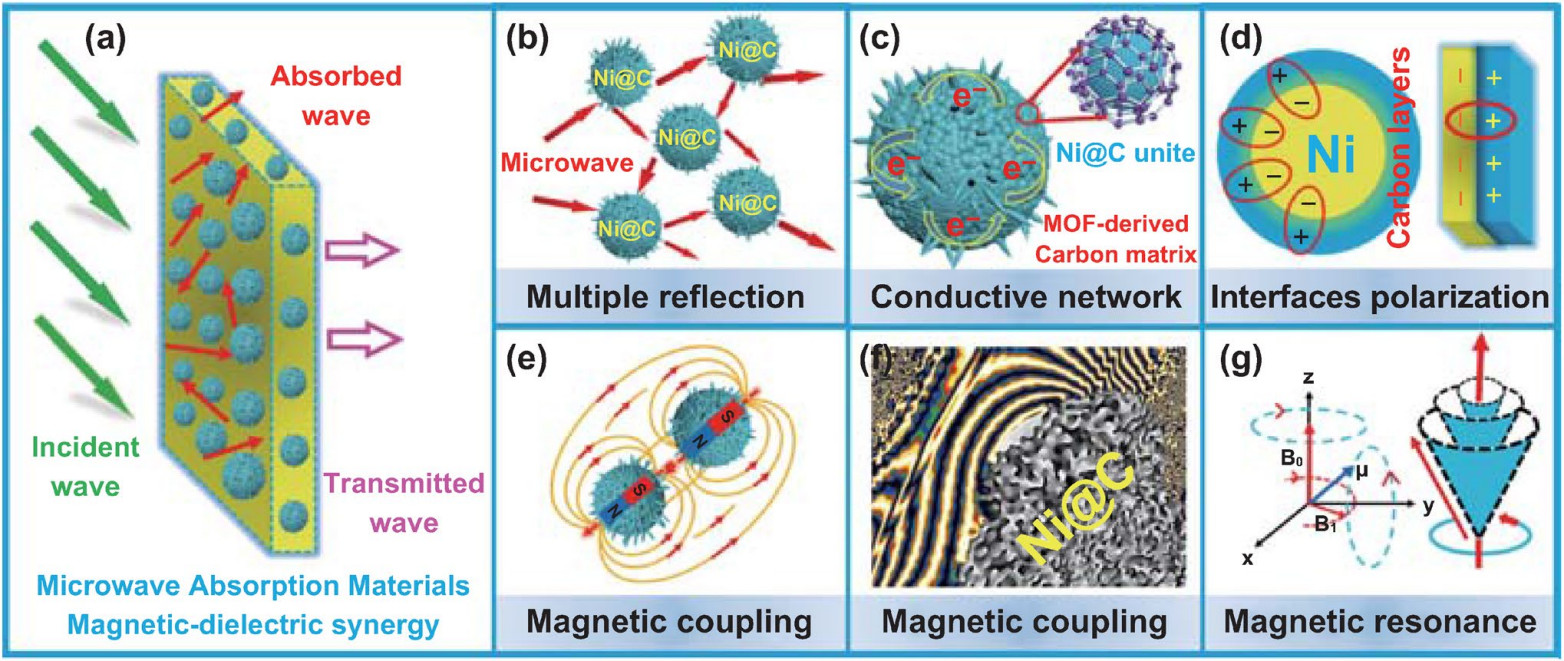
**a** Illustration of the MA process, **b** multiple reflection, **c** conduction loss, **d** interfacial polarization, **e**, **f** magnetic coupling, and **g** magnetic resonance of the Ni@C absorber

To fully understand the intrinsic MA mechanism, unique morphology, electromagnetic parameters, and dissipation behaviors are analyzed in detail to discover electromagnetic wave energy conversion. Taking Ni@C as an example, the MA mechanism mainly involves the impedance matching, dielectric loss, magnetic coupling, and the synergy effect in this magnetic carbon system. It is widely accepted that the impedance matching feature is the principal condition, which determines whether electromagnetic waves can enter the material system [46]. Aiming to get the good impedance watching, the MA system needs to adjust the intrinsic conductivity and magnetic parameters as well as the microspheres structure. In genial, the electromagnetic wave has the fixed impedance value (*Z*_0_ ~ 377 Ω) when traveling in free space. For the Ni@C absorber, the matching behavior is charge by the complex permittivity and complex permeability reflecting the input characteristic impedance (*Z*_in_). As displayed in Fig. 7a, it means the microwave can easily penetrate synergy absorber system rather than reflect black to the free space. In the Ni@C composites, the balanced electromagnetic parameters are encouraged by the evenly distribution of the components, which is benefit to enhanced impedance matching. Simultaneously, porous Ni@C composite at micrometer scale can provide multiple reflection, increasing the transmission path of electromagnetic waves and boosting the energy dissipation (Fig. 7b).

As a magnetic–dielectric synergy MA composite, the dielectric loss of Ni@C composite mainly consists of two parts: one is the polarization loss, and the other is the conductive loss [47–51]. In the MOF-derived black MA powders, lots of nanoscale Ni@C core–shell particles assemble into the magnetic carbon Ni@C microsphere containing kinds of interfaces. Benefiting from the coordination effect between the metal nickel and the organic ligand, carbonized Ni–MOF keeps the initial morphology and the metal clusters are confined by the surrounding carbon layer. Therefore, plenty of heterostructure interfaces exist in the special MOF-derived Ni@C composite. Unique contacting interfacial areas are constructed by the metal Ni particle and the graphitized carbon component (Fig. 7d). Meanwhile, touching interfaces also can be observed in the MOF-derived carbon matrix after the annealing treatment; the carbon layers as shell not only wrap the Ni core but also touch the other neighboring carbon layers (Fig. 7c). Therefore, driven charges gradually gather at the region such as formed Ni–C and C–C interfaces; the accumulated positive and negative charge locates at one side of the interface, respectively, which is benefit to the enhanced interfacial polarization in the applied electromagnetic wave environment [52, 53]. Limited to the catalytic capability of reduced Ni particles, there are still disorder carbon and defects in the graphitized carbon matrix. Under the action of high-frequency microwave, the movement of dipole moments caused by the separation of positive and negative charges promotes the conversion of microwave energy. In addition, MOF-derived Ni–C absorber possesses the highest attenuation constant (ɑ), which represents the strongest dissipation compared with other Ni@C composites (Fig. S5).

Dielectric loss behavior is high association with the electrical conductivity (*σ*) of the Ni@C absorber, which plays a key role to affect the storage and loss ability toward the electromagnetic wave energy. According to the Debye theory, the relationship between the complex permittivity (*ε*′, *ε*″) and intrinsic conductivity (*σ*) can be explained as follows: [54, 55]:

3 $$\varepsilon^{\prime} = \varepsilon_{\infty } + \frac{{\varepsilon_{\text{s}} - \varepsilon_{\infty } }}{{1 + \left( {\omega \tau } \right)^{2} }}$$

4 $$\varepsilon^{\prime\prime} = \frac{{\varepsilon_{\text{s}} + \varepsilon_{\infty } }}{{1 + \left( {\omega \tau } \right)^{2} }}\omega \tau + \frac{\sigma }{{\omega \varepsilon_{0} }}$$

where the *ε*′ and *ε*″ are the real and imaginary part of complex permittivity, *ε*_s_ and *ε*_∞_ represent the static and infinite permittivity, *ω* denotes the angular frequency, τ is the temperature-dependent relaxation time, *σ* is the conductivity. Based on the mentioned equations, the storage ability (*ε*″) is proportional to the conductivity (*σ*). The higher electron migration rate is favor to the strengthen microwave energy conversion [56]. For the Ni@C microspheres, enhanced electron migration can be obtained after converted from the Ni–MOF into magnetic carbon composite. In the carbothermal reduction process, metal Ni particles first form to promote the conductivity and the imaginary part (*ε*″), which is necessary to microwave energy dissipation. Meanwhile, the formed Ni clusters work as the catalyst further encouraging the graphitized conversion of the carbon-containing organic ligands. MOF-derived Ni@C composites are assembled by the metal nickel particles and the high conductivity carbon matrix. Graphitized carbon layers with *sp*^2^ arrangement can offer ballistic electron transport path, facilitating rapid electron migration. Therefore, both the carbon shell and derived carbon substrate not only can construct the unique conductive network but also offer powerful conduction loss (Fig. 7c).

**Fig. 8:**
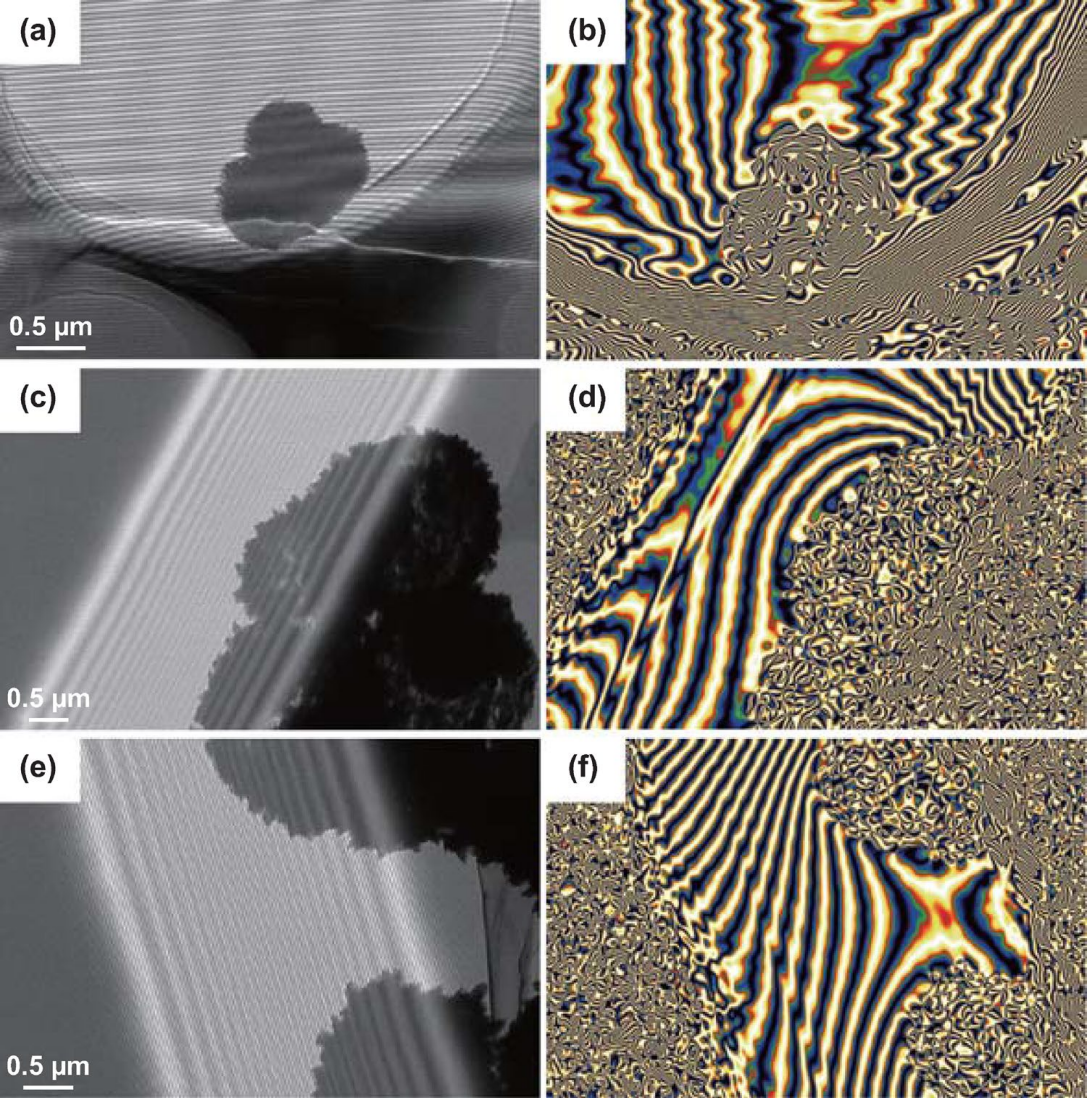
**a**, **c**, **e** Holography images and **b**, **d**, **f** reconstructed magnetic field line distribution of Ni@C composite

Equally important, the Ni@C composite can provide excellent magnetic properties, special magnetic coupling effect, and boosted magnetic loss behavior [57]. As displayed in Fig. 2c, the Ni@C composite owns the highest saturation magnetization (*M*s: 138.5 emu g^−1^) and lowest coercivity value, which is benefit to the promotion of imaginary part (*μ*″) and magnetic responding ability. In order to observe the intrinsic magnetic characteristic of the Ni@C microsphere, an off-axis electronic holography is used to discover the magnetic field line distribution, which offers the powerful evidence for the understanding of the magnetic loss mechanism [58, 59]. Clearly, the strong magnetic Ni@C absorber can release its own magnetic field line from the microspheres’ surfaces as shown in Fig. 8. The region of magnetic field line distribution is far beyond the volume of the material itself, which is never happened in the pure dielectric MA system (Fig. 8a, b). Magnetic–dielectric Ni@C absorber not only shows the inner magnetic loss in the microsphere structure, but also expands the magnetic responding scale, demonstrating its superiority as a MA material. Meanwhile, the electromagnetic energy attenuation can be accelerated because of the existence of the magnetic coupling phenomenon (Fig. 7e). As shown in Fig. 8c, d, neighboring Ni@C microspheres display an integral distribution of magnetic field lines rather than a single individual. The appearance of magnetic coupling further verifies the whole and promotes magnetic properties, playing an important dissipation mechanism (Fig. 7f). Meanwhile, due to the interaction between the magnetic Ni@C particles, the magnetic strength of the regions between the microspheres can still be increased even if there is no contact between the particles (Fig. 8e, f). These visual holography images and reconstructed magnetic flux line pictures directly prove the strong magnetic responding ability and magnetic coupling effect in the Ni@C system. When applied the high-frequency electromagnetic wave at 2–18 GHz, magnetic MA material of Ni@C microsphere embodies different feedbacks at given frequency region. Natural resonance dominates the main mechanism to attenuate electromagnetic wave energy under ~ 8 GHz. As frequency increases, magnetic loss behavior depends on the exchange resonance at the 10–18 GHz [60–62]. The mentioned ferromagnetic resonance contributes to the enhanced microwave energy conversion (Fig. 7g).

Considering the impedance matching feature and microwave dissipation ability, magnetic–dielectric Ni@C absorber exhibits excellent matching property and high-performance MA. The maximum *RL* of MOF-derived Ni@C composite up to − 59.5 dB at 6.0 GHz at low adding mass of 25% and the EAB can cover 4.7 GHz from 9.9 to 14.6 GHz. Different with the Ni@C absorber, other porous Ni@C composites need to increase the content of effective materials to achieve same excellent MA performance. When the adding mass up to 40%, the maximum *RL* of Ni_0.8_Co_0.2_@C composite reaches − 39.3 dB at 5.7 GHz and the EAB up to 4.8 GHz from 12.8 to 17.6 GHz. MOF-derived Ni@C absorber possess the high MA intensity and wider EAB, showing its potential as MA material in the practical application. As discussed above, the outstanding MA performance of MOF-derived Ni@C can be attributed to the good impedance, strong magnetic and dielectric loss, involving enhanced polarization dissipation, boosted conduction loss, and advanced magnetic coupling effect. As a result, MOF-derived magnetic–dielectric composites provide eye-catching inspiration for fabricating high effective microwave absorption materials.

## Conclusion

In summary, MOF-derived tunable Ni@C composites with unique nano–microstructure are successfully fabricated via solvothermal reaction and carbothermal reduction process. As expected, magnetic–dielectric synergy Ni@C composites, as MA materials, possess the strongest RL value − 59.5 dB (4.5 mm, 25% adding mass) and the effective absorption frequency covered as wide as 4.7 GHz ranging from 9.9 to 14.6 GHz at 2.5 mm. By increasing the absorber content, the MA capacity also can be boosted for other Ni_1−*x*_Co_*x*_@Carbon composites. The maximum RL of Ni_0.8_Co_0.2_@C composite reaches − 39.3 dB at 5.7 GHz and the EAB up to 4.8 GHz from 12.8 to 17.6 GHz. Benefiting from the coordination, carbonized bimetallic Ni–Co–MOF maintains its initial skeleton and transforms into magnetic carbon MA material with good impedance matching and multiple loss mechanism, involving interfacial polarization, conduction loss, and magnetic dissipation. Intrinsic dielectric loss ability and magnetic responding capacity of the Ni@C absorbers are significantly promoted to promote the electromagnetic wave energy conversion. Therefore, this study highlights that the MOF-derived magnetic/dielectric synergy Ni@C composites with unique nano–microstructure can be a lightweight, wideband, and highly efficient MA materials.

## Electronic supplementary material

Below is the link to the electronic supplementary material.

Supplementary material 1 (PDF 1011 kb)
