# Supplementary material for: MOF-Derived Ni1−xCox@Carbon with Tunable Nano–Microstructure as Lightweight and Highly Efficient Electromagnetic Wave Absorber
Source: Nanomicro Lett. 2020 Jul 15;12:150. doi: 10.1007/s40820-020-00488-0 (PMC7770844; doi:10.1007/s40820-020-00488-0)
Supplement: Supplementary file 1 — Supplementary material 1 (PDF 1011 kb) [file 40820_2020_488_MOESM1_ESM.pdf]

Supporting Information for

MOF-Derived $Ni_{1-x}Co_x$@Carbon with Tunable Nano-Micro Structure as Lightweight and Highly Efficient Electromagnetic Wave Absorber

Lei Wang[1], Mengqiu Huang[1], Xuefeng Yu[1], Wenbin You[1], Jie Zhang[1], Xianhu Liu[2], Min Wang[1], Renchao Che[1, *]

[1]Laboratory of Advanced Materials, Department of Materials Science and Collaborative Innovation Center of Chemistry for Energy Materials (iChem), Fudan University, Shanghai 200438, People's Republic of China

[2]Key Laboratory of Materials Processing and Mold (Zhengzhou University), Ministry of Education, Zhengzhou 450002, People's Republic of China

*Corresponding author. E-mail: rcche@fudan.edu.cn (Renchao Che)

Supplementary Table and Figures

Table S1 Information of MOF-derived $Ni_{1-x}Co_x$@Carbon composites

Sample	Size (um)	I_D/I_G	*M*s (emu/g)
Ni@C	1.5~2	0.92	138.5
$Ni_{0.8}Co_{0.2}$@C	3	1.06	131.4
$Ni_{0.5}Co_{0.5}$@C	8	0.95	121.1
$Ni_{0.2}Co_{0.8}$@C	10	0.98	100.7
CoO@C	30	-	47.2

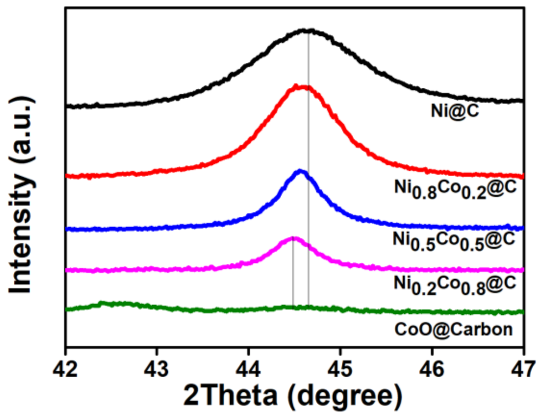

Fig. S1 XRD of obtained $Ni_{1-x}Co_x$@Carbon composites

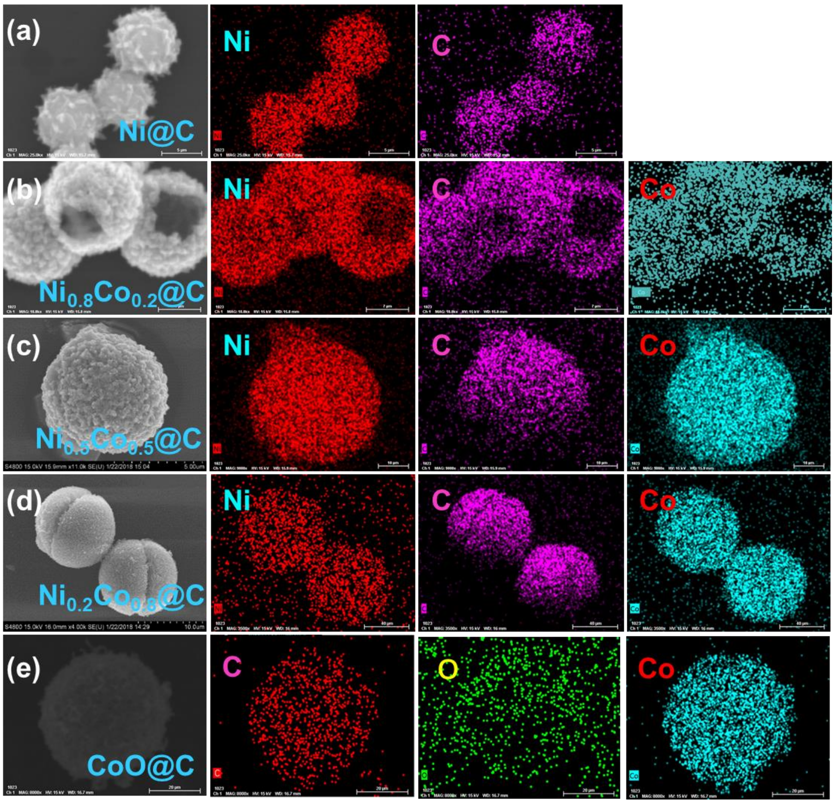

Fig. S2 Elements mapping distribution of (**a**) Ni@C, (**b**) $Ni_{0.8}Co_{0.2}$@C, (**c**) $Ni_{0.5}Co_{0.5}$@C, (**d**) $Ni_{0.2}Co_{0.8}$@C, and (**e**) CoO@C composites.

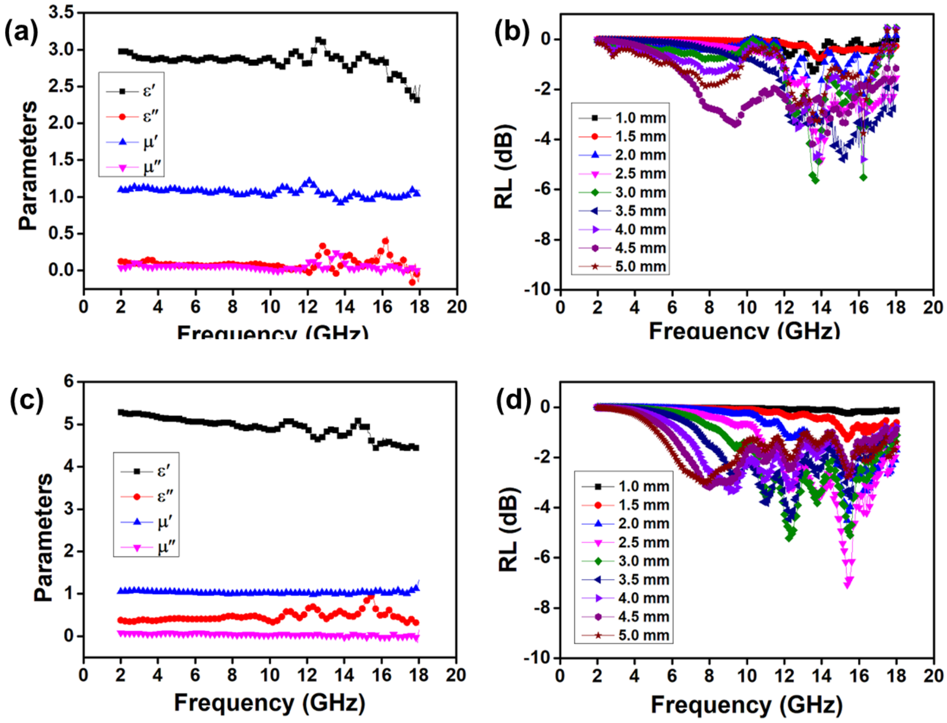

Fig. S3 Electromagnetic parameters (**a, c**) and microwave absorption (**b, d**) of obtained CoO@C composites at 25% and 40% mass adding, respectively

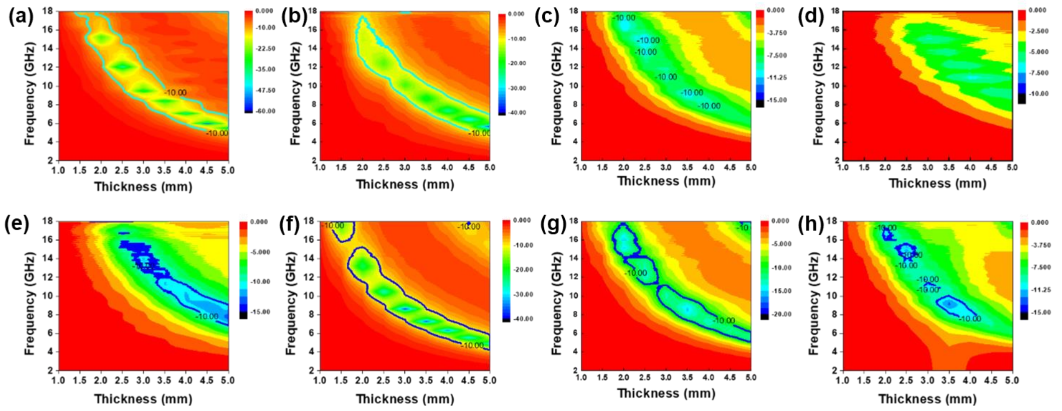

Fig. S4 Efficient absorption areas of (**a, e**) Ni@C, (**b, f**) $Ni_{0.8}Co_{0.2}$@C, (**c, g**) $Ni_{0.5}Co_{0.5}$@C, (**d, h**) and $Ni_{0.2}Co_{0.8}$@C composites at 25% and 40% mass adding, respectively

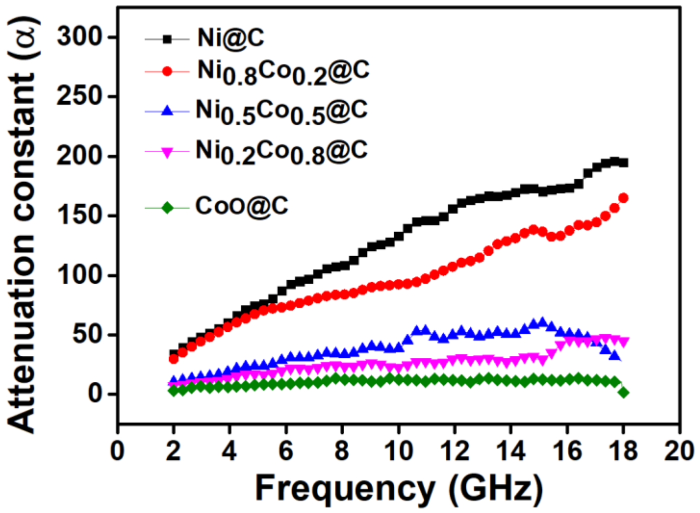

Fig. S5 Attenuation constant α of MOF-derived Ni1-xCox@C composites
